# Genomic and transcriptomic analysis of *Candida intermedia* reveals the genetic determinants for its xylose-converting capacity

**DOI:** 10.1186/s13068-020-1663-9

**Published:** 2020-03-12

**Authors:** Cecilia Geijer, Fábio Faria-Oliveira, Antonio D. Moreno, Simon Stenberg, Scott Mazurkewich, Lisbeth Olsson

**Affiliations:** 1 Division of Industrial Biotechnology, Department of Biology and Biological Engineering, Chalmers University of Technology, Gothenburg, Sweden; 2 Present Address: Biofuels Unit, Department of Energy, CIEMAT, Madrid, Spain; 3 Department of Chemistry and Molecular Biology, University of Gothenburg, Gothenburg, Sweden

**Keywords:** *Saccharomyces cerevisiae*, Xylose utilization, Complete genome sequence, RNA-Seq, Xylose/aldose reductase, NADH-preferring xylose reductase, Biofuels, Pentose metabolism

## Abstract

**Background:**

An economically viable production of biofuels and biochemicals from lignocellulose requires microorganisms that can readily convert both the cellulosic and hemicellulosic fractions into product. The yeast *Candida intermedia* displays a high capacity for uptake and conversion of several lignocellulosic sugars including the abundant pentose d-xylose, an underutilized carbon source since most industrially relevant microorganisms cannot naturally ferment it. Thus, *C. intermedia* constitutes an important source of knowledge and genetic information that could be transferred to industrial microorganisms such as *Saccharomyces cerevisiae* to improve their capacity to ferment lignocellulose-derived xylose.

**Results:**

To understand the genetic determinants that underlie the metabolic properties of *C. intermedia*, we sequenced the genomes of both the in-house-isolated strain CBS 141442 and the reference strain PYCC 4715. De novo genome assembly and subsequent analysis revealed *C. intermedia* to be a haploid species belonging to the CTG clade of *ascomycetous* yeasts. The two strains have highly similar genome sizes and number of protein-encoding genes, but they differ on the chromosomal level due to numerous translocations of large and small genomic segments. The transcriptional profiles for CBS 141442 grown in medium with either high or low concentrations of glucose and xylose were determined through RNA-sequencing analysis, revealing distinct clusters of co-regulated genes in response to different specific growth rates, carbon sources and osmotic stress. Analysis of the genomic and transcriptomic data also identified multiple xylose reductases, one of which displayed dual NADH/NADPH co-factor specificity that likely plays an important role for co-factor recycling during xylose fermentation.

**Conclusions:**

In the present study, we performed the first genomic and transcriptomic analysis of *C. intermedia* and identified several novel genes for conversion of xylose. Together the results provide insights into the mechanisms underlying saccharide utilization in *C. intermedia* and reveal potential target genes to aid in xylose fermentation in *S. cerevisiae*.

## Background

In a sustainable society that relies on a circular bio-based economy, lignocellulosic biomasses such as energy crops, agricultural and forestry residues will be important starting materials for the production of fuels, chemicals and materials. Lignocellulosic biomass that consists of 35–50% cellulose, 20–35% hemicelluloses and 15–20% lignin must be pre-treated and hydrolysed to obtain the monomeric sugars that can be converted into desired product(s) through microbial fermentation (review in [1]). The hemicellulosic pentoses d-xylose and l-arabinose are currently underutilized resources, as many of the industrially relevant microorganisms (e.g. *Saccharomyces cerevisiae*) lack the ability to ferment these sugars. For economically viable fermentation-based production processes, discovery and development of microorganisms that can readily ferment all lignocellulosic sugars is of foremost importance [2].

Xylose fermentation has attracted a lot of research attention, as xylose is the second, after glucose, most abundant monomeric sugar in lignocellulosic biomass [2, 3]. A number of native xylose-fermenting yeasts (e.g. *Scheffersomyces stipitis*, *Spathaspora passalidarum, Sugiyamaella lignohabitans*, and *Candida tenuis*) have been identified and characterized to various degrees in terms of metabolism, physiology, genomics and proteomics. Their usefulness for lignocellulosic conversion processes has been explored, but as cell factories they are limited by their (i) dependence on micro-aerobic conditions for fermentative metabolism, (ii) low tolerance to lignocellulosic inhibitors and (iii) poor overall fermentation titers [4, 5]. As an alternative, xylose-fermenting yeasts can serve as a source of knowledge and genes for xylose uptake and assimilation which may be transferrable to other microorganisms including *S. cerevisiae* that are better suited as industrial workhorses.

Numerous *S. cerevisiae* strains capable of xylose fermentation have been engineered through heterologous expression of either the xylose oxidoreductive pathway (xylose reductases (XR) and xylitol dehydrogenases (XDH)) donated from various xylose-utilizing yeasts, or the xylose isomerase (XI) pathway of either bacterial or fungal origin [3, 6]. Despite the many attempts to optimize the xylose metabolic pathway fluxes by Adaptive Laboratory Evolution (ALE) and metabolic engineering [7–10] and as reviewed in [11], several physiological issues remain that prevent xylose from being fermented as efficiently as glucose in this species. The primary reason for the low xylose-fermenting capacity of *S. cerevisiae* is that it lacks an efficient and specific xylose transport system, which hampers the metabolite’s uptake and the cell’s subsequent metabolic flux. Instead, xylose enters the cell through hexose transporters (in particular Hxt1, Hxt4, and Hxt7), which all have 100-fold greater affinities for glucose over xylose [12]. Moreover, transporter discrimination in combination with glucose repression mechanisms make *S. cerevisiae* ferment glucose prior to xylose in mixed sugar conditions [13]. This is problematic in fermentations of lignocellulose hydrolysates containing inhibitory compounds, as cells are more sensitive to these substances during xylose-conversion than during glucose-conversion, which leads to stalled fermentations in the xylose phase [14]. Simultaneous saccharification and co-fermentation (SSCF) setups where glucose/xylose co-fermentation is key for an efficient process are also severely limited by suboptimal utilization of xylose in recombinant strains of *S. cerevisiae* [15]. Lastly, *S. cerevisiae* strains relying on the co-factor-dependent XR/XDH system often stall during xylose fermentation as incomplete co-factor recycling leads to accumulation of xylitol and ultimately results in a lower overall product yield [16]. The incomplete co-factor recycling results from the majority of XRs having a preference for NADPH over NADH while XDHs use NAD^+^, which ultimately leads to insufficient amounts of NAD^+^ being regenerated [11, 17].

In a previous ALE experiment using repetitive batch cultures with increasing concentrations of the liquid fraction of a lignocellulosic hydrolysate, we sought to improve the xylose-fermentative capacity of an industrial strain of *S. cerevisiae* equipped with the XR/XDH genes from *S. stipitis*. To our surprise, we learned that the cell population had been contaminated with a xylose-utilizing yeast species that managed to compete with *S. cerevisiae* in the xylose-rich, highly inhibiting environment that characterizes a typical lignocellulosic hydrolysate. Several clones were isolated throughout the experiment, and the contaminants were identified to the species level by PCR amplification and sequencing of the internal transcribed spacer (ITS) regions and the D1–D2 region of the large-subunit RNA gene [18, 19]. The sequences were 98–100% (ITS) and 99–100% (D1–D2) identical with a large number (> 20) of *Candida intermedia* strains. As strains with higher than 99% identity in the D2 domain are considered the same or sister species [19], we concluded that the isolates were of *C. intermedia* origin.

*C. intermedia* is an interesting species for industrial conversion of lignocellulosic biomass, as it possesses many of the traits that xylose-fermenting *S. cerevisiae* strains lack. Its specific growth rate on xylose outcompetes most other yeast species [20], and two different *C. intermedia* xylose-transporters have previously been identified and characterized; the glucose/xylose facilitator 1 Gxf1, which facilitates high-capacity, low-affinity diffusion of glucose and xylose, and the glucose/xylose symporter 1 Gxs1 which is a high-affinity glucose/xylose-proton symporter [20–25]. *C. intermedia* also expresses an XR with preference for both NADH and NAPDH [26], which can alleviate co-factor imbalance during xylose fermentation. Moreover, we have shown in a previous study that a clone isolated from the end population of the ALE experiment, CBS 141442, is capable of glucose and xylose co-fermentation in wheat straw hydrolysates [14].

In the present study, the aim was to elucidate the genetic features of *C. intermedia*, which form the basis for its industrially interesting traits. We report here on the complete genome sequence of *C. intermedia,* its transcriptional response to xylose and glucose and the identification of several novel genes with potential to contribute to xylose conversion in lignocellulose-based production of biofuels and biochemicals.

## Results and discussion

### De novo genome sequencing of *C. intermedia*

To enable analysis of the in-house isolated *C. intermedia* strain CBS 141442 on a genomic and molecular level, we set out to determine its genome sequence. For comparative reasons we also sequenced the *C. intermedia* strain PYCC 4715, a strain previously characterized in terms of xylose uptake and assimilation [20]. The genome of CBS 141442 was sequenced to 61X coverage and assembled into nine chromosomal contigs and a mitochondrial contig. Essentially, each chromosomal contig resembles one chromosome, except the largest chromosome that consists of three contigs with the middle one being flanked by ribosomal repeats, resulting in seven chromosomes in total. The genome of PYCC 4715 was sequenced to 32× coverage, and the 27 contigs were reference-guided with the CBS 141442 chromosomes as a backbone to create full-length chromosomes. The genome sizes were summed up to 13.2 Mbp (CBS 141442) and 13.1 Mbp (PYCC 41715), both with a mean G+C content of 43.5%. A total of 5936 and 6073 protein coding genes were identified for CBS 141442 and PYCC 4715, respectively, and genome completeness according to CEGMA was above 95% for both strains (Table 1) [27]. Overall, we can conclude that the two complete *C. intermedia* genomes are highly similar to each other in terms of size and protein coding genes, and the numbers correlate well with other sequenced *ascomycetous* yeast species [28].

**Table 1:** *C. intermedia* genomic features

Assembly and protein coding genes	CBS 141442	PYCC 4715
Genome size (Mbp)	13.2	13.1
GC content (%)	43.5	43.5
No. of contigs	9	27
Contig N50 (Mbp)	1.6	0.8
Completeness (CEGMA analysis) (%)	95.2	95.6
Chromosomes	7	7
No. of protein coding genes	5936	6073
No. of protein coding genes with functional annotation	4917	4937
Average coding DNA sequence length	1489	1273
Fraction of genome covered by genes (%)	67.9	67.2

### Genetic distance and genome synteny

**Fig. 1:**
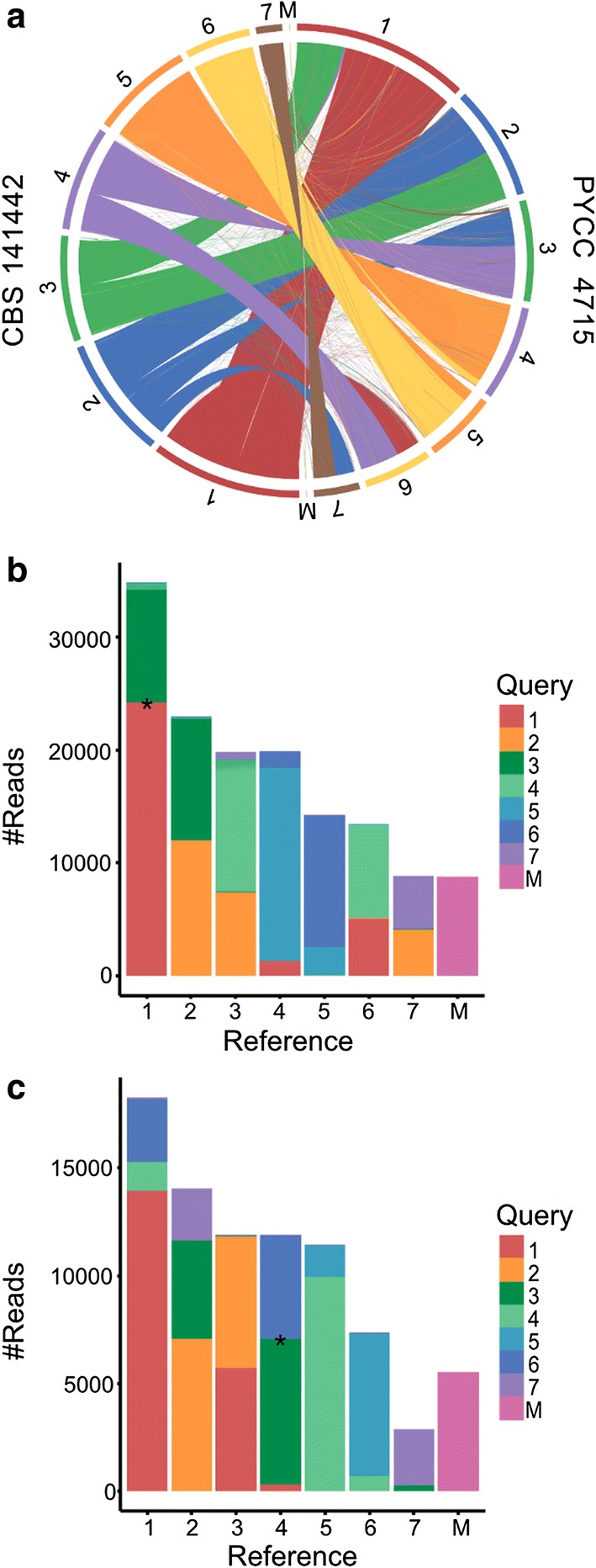
Genome differences due to numerous chromosomal translocations. **a** Circos plot of the syntenic block translocations between chromosomes in the two *C. intermedia* strains. The chromosomes are arranged in a circle, and each aligned segment was drawn as a ribbon from the chromosomes of CBS 141442 to PYCC 4715, where each CBS 141442 chromosome is marked with a unique colour to clearly visualize the genomic rearrangements. **b**, **c** Reciprocal read mapping, where **b** displays the CBS 141442 genome reads coloured according to which chromosome they belong to in the CBS 141442 assembly, and mapped to the PYCC 4715 chromosomes, and **c** marked reads from PYCC 4715 mapped to the CBS 141442 assembly. *Breakpoints confirmed with PCR

To determine the genetic distance between the two strains on a genome level, the average nucleotide identity (ANI) was calculated using both BLAST and MUMmer as search engines [29]. The sequence identity between the two assemblies was 97% for both runs, and cut-off scores of > 95% confirm that they belong to the same species [30]. We also determined the chromosomal map conservation between the strains. For visualization of the results, the two sets of chromosomes were placed in a circle with the ribbon tracks displaying synteny blocks for the whole genome alignment (Fig. 1a). Although the overall order of genes is highly conserved, every chromosome displays 1 or 2 translocations of large genome blocks, resulting in that all CBS 141442 chromosomes are composed of a mosaic pattern with 2–3 conserved syntenic segments of individual PYCC 4715 chromosomes, and vice versa. In addition, numerous smaller syntenic blocks are translocated throughout the genomes in a non-reciprocal manner. As the chromosomes are numbered in order of size with the largest labelled 1, the large rearrangements result in that the CBS 141442’s chromosome 4, 5 and 6 are labelled as chromosome 5, 6 and 4 in PYCC 4715. Reciprocal read mapping was used as an alternative approach to assess the translocations, which confirmed the results of the full genome alignment (Fig. 1b, c). Finally, two randomly chosen chromosomal fusion points were confirmed by PCR (indicated by * in Fig. 1b, c). These results collectively indicate that the translocations observed in the different strains are real and not due to errors in genome assembly.

For most non-conventional yeast species, knowledge of sequence identity and genome synteny of different strains is sparse. Typically, only one or a very small number of strains per species have been sequenced, and most isolated strains are classified using ITS/D1–D2 sequences alone. For model organisms such as *S. cerevisiae* where multiple strains have been sequenced, the relevance of genome diversity in natural and domestic populations for adaptation to new and changing environments has been demonstrated [28]. We can only speculate about the reason for the chromosomal translocations observed in the two *C. intermedia* genomes. The translocations might shed light on a common phenomenon among *C. intermedia* and *ascomycetous* yeast as a strategy of survival and/or the continuous process of speciation [31]. Alternatively, these translocations could be a result of CBS 141442 being isolated from a highly challenging condition of repetitive batch cultures with toxic wheat straw hydrolysates [14], as such evolutionary engineering schemes are prone to generate genome alternations [32].

### Phylogenetic relationship of *C. intermedia* with other yeasts

**Fig. 2:**
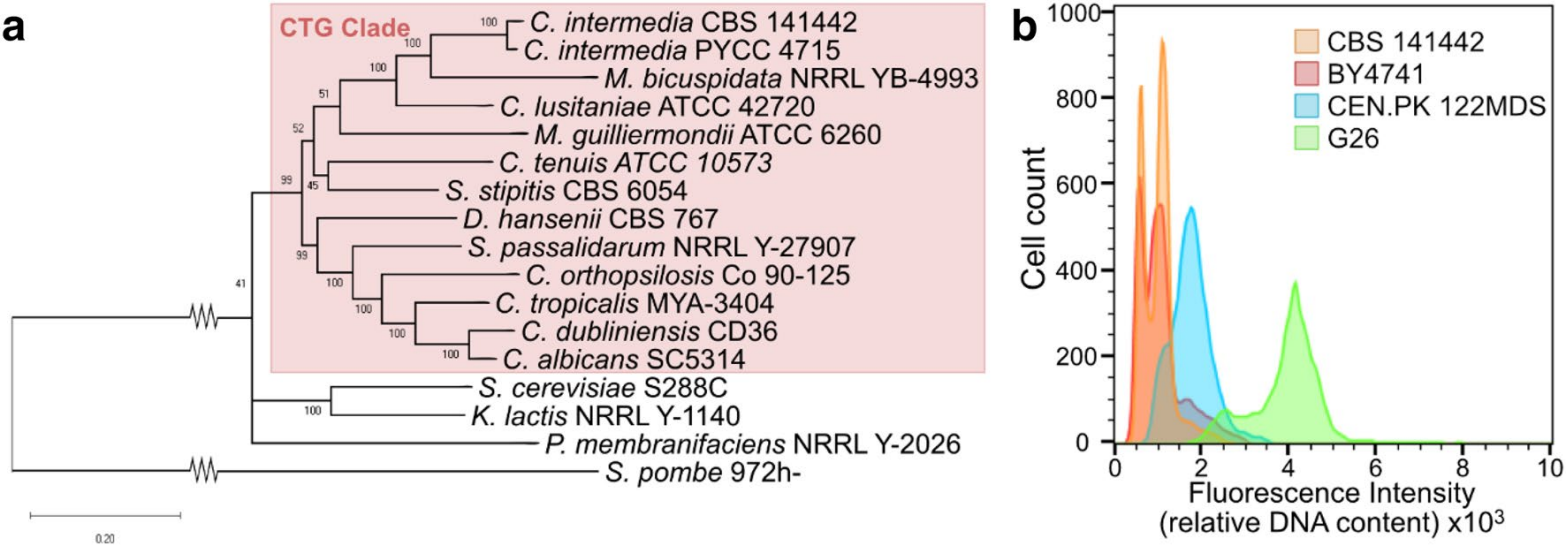
Phylogenetic placement and ploidy determination of *C. intermedia*. **a** Phylogenetic tree of *C. intermedia* CBS 141442 and PYCC 4715 and 15 other *ascomycetous* yeasts. The molecular phylogenetic analysis was based on ten protein-encoding genes using the maximum likelihood method. The tree is drawn to scale, with branch lengths measured in the number of substitutions per site. The numbers at each branch indicate bootstrap values, and the CTG branch is highlighted. **b** Ploidy determination of *C. intermedia* CBS 141442 by flow cytometry. Three *S. cerevisiae* strains were used as reference; haploid BY4741, diploid CEN.PK and tetraploid 122 MDS

To determine the evolutionary relationship of the two *C. intermedia* strains with other yeasts of industrial and clinical relevance, we selected 15 genome sequenced *ascomycetous* yeast strains with industrial or clinical importance and performed a phylogenetic analysis. The resulting tree shows that the two *C. intermedia* strains cluster together with species within the CTG clade (Fig. 2a), in good correlation with previous work based on ITS/D1–D2 sequence alignments alone [33]. The webserver Bagheera [34] was used to confirm CUG codon usage and the haploid identity of *C. intermedia CBS 141442* was determined using flow cytometry analysis (Fig. 2b).

Many members of the CTG clade are commensal with humans or other host organisms, and can emerge as opportunistic pathogens (*Candida albicans, Candida tropicalis* [35, 36]). Other species, such as *S. stipitis* and *S. passalidarum*, have adapted to living in the guts of wood-consuming beetles and are capable of fermenting xylose, yet others are associated with marine environments (*Metschnikowia bicuspidata* and *Debaryomyces hansenii*) or plants and soil (*Clavispora lusitaniae*) [37–42]. The biological niche of *C. intermedia* has not been thoroughly investigated, but strains deposited in different databases have been isolated from a range of environmental sources including fruits and flowers, dairy products and humans (listed in Additional file 1). From the information collected, we can conclude that *C. intermedia* can be found in diverse habitats around the world, including also a number of xylose-rich substrates such as decaying forest residues and biomass hydrolysates, where *C. intermedia’*s high capacity of xylose assimilation could be an advantageous trait.

### Transcriptional profiles of *C. intermedia* grown in xylose and glucose containing media

**Fig. 3:**
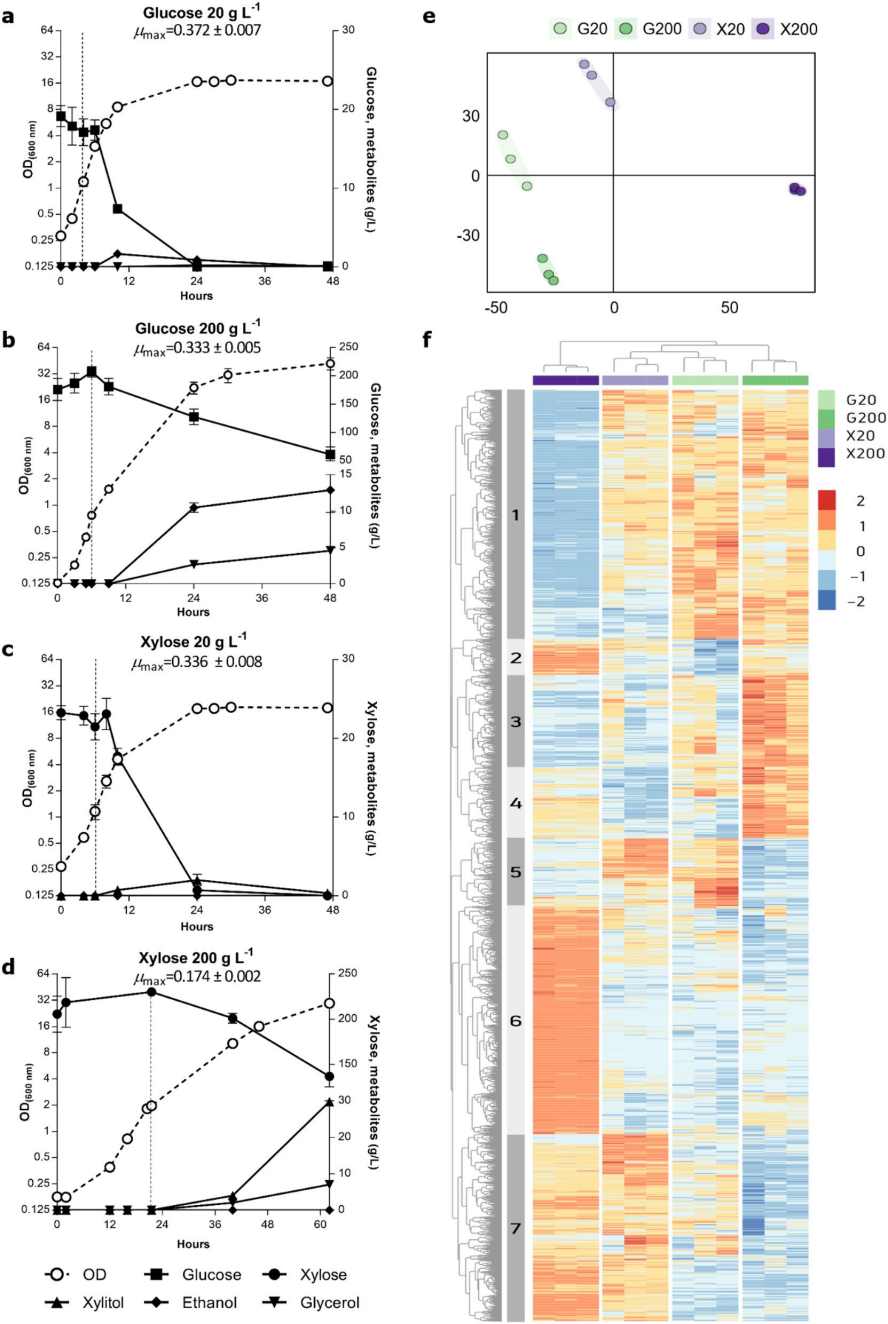
*C. intermedia* growth, sugar consumption and transcriptional profiles in media with different sugar compositions. **a**–**d** Growth (OD_600_) and extracellular sugar and metabolite concentrations over time. µmax represents the maximum growth rate (h^−1^) of the cultures. Vertical dashed lines represent the sampling time points for RNA-seq. **e** MDS plot of gene expression of 12 RNA-seq libraries. Samples cluster by carbon source and concentration. **f** Heatmap plot of differently expressed genes using the hierarchical clustering method, displaying seven main clusters. Each row of the heatmap represents the z-score transformed log 2 values of one differentially expressed gene across all samples (blue, low expression; red, high expression). Colours above the heatmap indicate carbon source and concentration (G20—glucose 20 g L^−1^; G200—glucose 200 g L^−1^; X20—xylose 20 g L^−1^; X20—xylose 200 g L^−1^). The dendrogram displays how the samples cluster based on their differential expression patterns

To identify the genes responsible for utilization of xylose in *C. intermedia*, we performed a transcriptome analysis using RNA-sequencing (RNA-seq) technology. The CBS 141442 strain was cultivated in bioreactors in medium containing xylose or glucose (as reference), both at a concentration of 20 g L^−1^ to promote rapid cell growth and at 200 g L^−1^ to assess the transcriptional reponse to high sugar concentrations encountered during industrial fermentations. The strain displayed similar specific growth rates in glucose and xylose at 20 g L^−1^ (0.37 and 0.34 h^−1^, respectively) (Fig. 3a, c), which correlates well with what has been shown previously for *C. intermedia* PYCC 4715 and FL023 [20, 43]. The specific growth rate was only slightly affected in 200 g L^−1^ of glucose (0.33 h^−1^) (Fig. 3b), confirming our previous experiments showing that *C. intermedia* is a highly osmotolerant yeast (unpublished results). However, the yeast was clearly challenged in 200 g L^−1^ of xylose as judged by the drop in specific growth to 0.17 h^−1^ (Fig. 3d), suggesting that high concentrations of xylose exert an osmotic stress on the cells. This is in line with previous observations for *C. intermedia,* as well as for other yeasts, where cells grown in xylose are comparatively more sensitive to stress factors in the environment than glucose-grown cells, possibly due to lower intracellular ATP levels [14, 44–49]. Moreover, *C. intermedia* switched from a respiratory to fermentative metabolism when oxygen became limited in the cultures (due to fixed stirring and sparging settings in the experimental design), evidenced by the formation of ethanol and the by-products glycerol and xylitol (Fig. 3a–d). This is indicative of Crabtree-negative yeasts, where the onset of fermentation is regulated by a decrease in oxygen levels [50].

The transition from respiratory to fermentative growth in Crabtree-negative yeasts is characterized by a significant change in transcription [51]. Thus, to enable comparison of the overall transcriptional profiles for the different conditions in a straightforward way, samples for RNA-seq were collected in mid-exponential, respiratory phase at maximum growth rate when no metabolites were accumulating (dotted lines Fig. 3a–d), and purified mRNA was sequenced as outlined in the materials and methods section. The resulting RNA-seq datasets were used in an unsupervised hierarchical clustering of the 5761 expressed genes across the four growth conditions. The multi-dimensional scaling (MDS) plot shows that the triplicates for each condition cluster together, and that the gene expression profiles from the cells grown in 20 and 200 g L^−1^ of glucose and 20 g L^−1^ of xylose are more similar to each other than to cells grown in 200 g L^−1^ of xylose (Fig. 3e). This difference is consistent with the observed slower specific growth rate in 200 g L^−1^ of xylose as discussed above. The trend is also demonstrated very clearly in the heat map, which revealed 7 distinct, condition-dependent clusters of differentially expressed genes and enriched gene ontology (GO) annotations (Fig. 3f, Additional file 2). In clusters 1, 2 and 6, the three fast-growing cultures displayed the opposite expression patterns from the culture with 200 g L^−1^ of xylose. Cluster 1 is composed of genes with an increased expression in cells with higher growth rates, and the annotations revealed an enrichment of genes associated with growth, including genes for organization of the cytoskeleton and the plasma membrane as well as RNA processing and translation. On the other hand, clusters 2 and 6 comprise genes that had higher expression in cells grown in 200 g L^−1^ of xylose and include a significant number of genes involved in regulation of transcription, protein phosphorylation and response to stress. Cluster 3 and 4 are markedly upregulated in 200 g L^−1^ of glucose and are composed of genes for translation and rRNA processing. Cluster 7 follows a carbon source-dependent transcription pattern and comprise genes upregulated in xylose conditions and downregulated in glucose-grown cells. Here, we found an enrichment of genes associated with carbohydrate metabolism and oxidation–reduction reactions, similar to what was previously shown for *S. stipitis* when grown in xylose [52]. To summarize, the major differences in transcriptional output seem to be correlated with the specific growth rate, osmotic stress and carbon source provided.

### Metabolic map of xylose and glucose utilization pathways

**Fig. 4:**
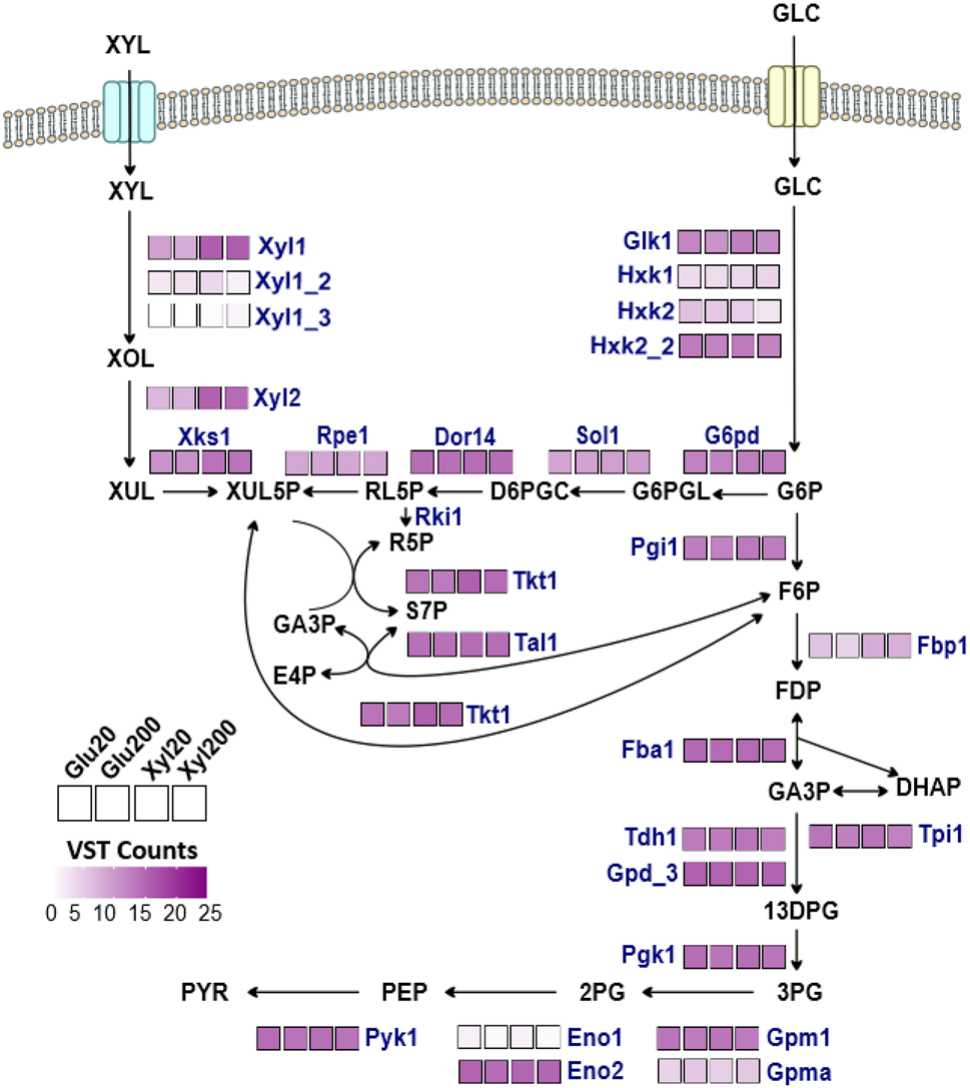
Expression profiles of genes encoding putative transporters and enzymes in the glucose and xylose metabolic pathways. Arrows represent enzyme reactions catalysed by the associated proteins. Normalized expression of enzyme-encoding genes is represented in all carbon sources and concentrations tested using variance-stabilized counts (VST). Enzymatic reactions substrates and products as follow: XYL—xylose; XOL—xylitol; XUL—xylulose; XUL5P—xylulose 5-phosphate; RL5P—ribulose 5-phosphate; R5P—ribose 5-phosphate; S7P—sedoheptulose 7-phosphate; GA3P—glyceraldehyde 3-phosphate; E4P—erythrose 4-phosphate; D6PGC—6-phosphogluconate; G6PGL—glucono 1,5-lactone 6-phosphate; G6P—glucose 6-phosphate; GLC—glucose; F6P—fructose 6-phosphate; FDP—fructose 1,6-biphosphate; DHAP—dihydroxyacetone phosphate; 13DPG—1,3-diphosphoglycerate; 3PG—3-phosphoglycerate; 2PG—2-phosphoglycerate; PEP—phosphoenolpyruvate; PYR—pyruvate. Accession numbers of all proteins are available in Additional file 4

The genomic and transcriptomic data collected allowed identification of genes encoding putative transporters and enzymes for xylose and glucose metabolism in *C. intermedia*. Accordingly, we scanned the CBS 141442 genome for genes encoding Major Facilitator Superfamily (MFS) sugar transporters and the RNA-seq dataset for the corresponding transcriptional profiles [53], and identified several orthologs to xylose transporters from other yeasts (Additional file 3) [21, 25, 54, 55]. Of particular interest were two novel genes encoding putative homologs to the previously characterized glucose/xylose transporters Gxf1 and Gxs1 (named Gxf1_2 and Gxs1_2, respectively). The four genes displayed different expression patterns which may indicate diverse physiological roles: whereas relatively high levels of *GXF1* and *GXF1_2* transcripts were observed in all conditions tested, *GXS1* expression appeared induced in xylose conditions and *GXS1_2* displayed a stable but low expression across all conditions assessed (Fig. 4). Further experimental characterization is needed to determine the transporters’ substrate specificities and their contribution to the total sugar uptake capacity of *C. intermedia*.

To identify the putative genes responsible for xylose and glucose catabolism in *C. intermedia*, we searched the CBS 141442 genome for genes encoding proteins with high homology to proteins involved in central metabolism in the *S. stipitis* CBS 6054 strain [56]. As expected, the genes ascribed a role in central carbon metabolism were strongly expressed in all four conditions, indicative of respiratory growth. The expression of genes encoding a putative XR (*XYL1*, EC 1.1.1.307), XDH (*XYL2*, EC 1.1.1.9) and xylulokinase (XK, *XKS1*, EC 2.7.1.17) was higher during growth on xylose than during growth on glucose, pointing towards that these genes are important for xylose utilization under the set conditions. Moreover, we found two additional putative XR genes, *XYL1_2* and *XYL1_3*, which share 62 and 75% identity to *XYL1*, respectively. *XYL1*_2 and *XYL1*_3 are minimally expressed in xylose (as well as in glucose-grown cells) (Fig. 4), suggesting minor roles for these enzymes during xylose growth in aerobic conditions.

### Characterization of the *C. intermedia* xylose reductases

The finding of multiple genes encoding putative XRs in the *C. intermedia* CBS 141442 genome is in line with the previous report that suggests *C. intermedia* to have two different enzymes with XR activity, one with preference for both NADH and NADPH and one with strict NADPH-dependence [26]. XRs with specificity for NADH are of particular interest as they can alleviate cofactor imbalance during xylose fermentation. To assess the cofactor preferences and substrate specificities of the three putative XRs identified, the genes were individually expressed in *S. cerevisiae* and characterized in vitro. The *S. stipitis XYL1_K270R* gene encoding an XR with dual cofactor specificity was also included as reference.

All of the *CiXYL1* gene products had XR activity using NADPH as cofactor and xylose as substrate, which confirmed that the enzymes were expressed and active in the *S. cerevisiae* host (Table 2). No, or minimal, reductase activity was observed using glucose as substrate indicating a specificity towards xylose (data not shown). When NADH was supplied as a cofactor, no XR activity was detected for CiXR1 and CiXR3, indicating that these enzymes have a high preference for NADPH over NADH. In contrast, both CiXR2 and SsXR_K270R could utilize NADH and NADPH with similar preferences. Taken together, we can conclude that *C. intermedia* possesses genes for three functional XRs and that CiXR2 is responsible for the previously reported NADH-dependent XR activity in this species [26].

**Table 2:** Kinetics of *C. intermedia* xylose reductases in crude cell extracts

	Specific activity (U (mg protein)^−1^)		
Construct	NADH	NADPH	Ratio NADH/NADPH
p426Ø	0.010 ± 0.079	0.067 ± 0.131	0.15
p426-SsXYL1_K270R	0.481 ± 0.046	0.721 ± 0.018	0.67
p426-CiXYL1	0.008 ± 0.008	0.351 ± 0.070	0.02
p426-CiXYL1_2	0.449 ± 0.016	0.456 ± 0.062	0.99
p426-CiXYL1_3	0.004 ± 0.128	0.312 ± 0.103	0.01

All of the enzymes were expressed in a *S. cerevisiae* strain deleted of the native aldose reductase *GRE3*. Thus, the basal cofactor reduction detected for the strain transformed with empty plasmid (0.010 and 0.067 U (mg protein^−1^) for NADH and NADPH, respectively) is due to spontaneous NAD(P)H oxidation over time, and/or to background enzymatic activities in the crude protein extract. Mean values ± SD from two independent experiments are represented, including cell cultivation, protein extraction and activity quantification

### Amino acids contributing to the co-factor preference of xylose reductases

**Fig. 5:**
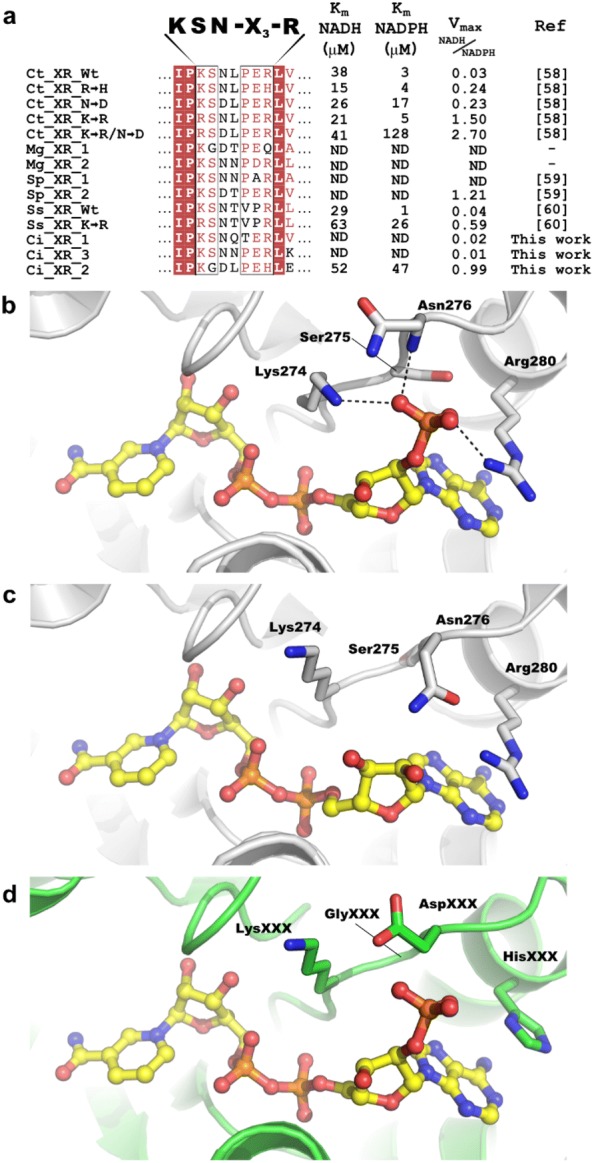
Comparison of characterized yeast XRs. **a** Excerpt of a multiple sequence alignment of yeast XRs showing key residues in the cofactor binding site. The KSN-X_3_-R protein motif implicated in NADPH specificity is highlighted, and mutations introduced into the genes are noted with arrows beside the species name. Identical residues are shown as white text on a red background and similar residues in red text on a white background. The kinetic parameters of each XR are shown where available alongside the parameters determined in this work. The species from which the XR originate are abbreviated as follows: Ct, *C. tenuis*; Mg, *M. guilliermondii*; Sp, *S. passalidarum*; Ss, *S. stipitis*; Ci, *C. intermedia*. **b** The cofactor binding site of *C. tenuis* XR in complex with NADP^+^ (PDB ID: 1k8c) and **c** with NAD^+^ (PDB ID: 1mi3). The hydrogen bonds stabilizing the NADP^+^ in the *C. tenuis* XR structure is shown with black dashes. **d** A homology model of the *C. intermedia* XR2 created with PHYRE [82] using the XR from *C. tenuis* as a template (PDB ID: 1k8c) to visualize the differences in the active site which may contribute to the lack NADP^+^ specificity

While the majority of characterized yeast XRs exclusively use NADPH as co-factor, the XR from *C. tenuis* displays dual co-factor specificity although with a 10-fold preference for NADPH over NADH. Solved structures of the apo- and holo-forms of CtXR show the co-factor binding pocket and how the enzyme can accommodate co-factors both with and without 2′-phosphate groups [57]. A conserved, key motif of KSN-X_(3)_-R found within the pocket aids in defining co-factor specificity, and numerous mutagenesis studies have revealed that changes in this motif can result in a loss of specificity of NADPH over NADH [58–60]. A multiple sequence alignment with XR yeast homologs revealed conservation of the KSN-X_(3)_-R motif in CiXR1 and CiXR3, consistent with their high preference for NADPH (Fig. 5a, Table 2).

In the structure where CtXR is bound to NADP^+^, the residues of the KSN-X_(3)_-R motif assist in co-factor binding by making hydrogen bonds with the 2′-phosphate moiety with a notable hydrogen bond contributed by the main chain amide of Asn276 (Fig. 5b). When CtXR is instead bound to NAD^+^, which lacks the 2′-phosphate moiety of NADP^+^, only minimal rearrangements are observed in the side chains of the KSN-X_(3)_-R motif, suggesting an active site fine-tuned for NADP^+^ (Fig. 5c).

In contrast to CtXR, CiXR2 showed similar affinity for both co-factors (Km of 52 µM for NADH and 47 µM for NADPH) (Fig. 5a). The residues in the binding-pocket motif in CiXR2 are KGD-X_(3)_-H (rather than the conserved KSN-X_(3)_-R), and these differences in the active site motif likely contribute to the enzyme’s lack of co-factor preference. A homology model of CiXR2, using CtXR as template, suggests that the N276D and S275G substitutions are key contributors to the differences in co-factor affinities observed for the two enzymes (Fig. 5d). The introduction of the acidic aspartate residue likely leads to electrostatic repulsion with the NADP^+^ 2′-phosphate, while the introduction of a neighbouring glycine would give an increased degree of flexibility for the region. This could lead to an alternate position of the Asp276 residue and a loss of the hydrogen bond made by the backbone amide with the 2′-phosphate.

The three substitutions in the CiXR2 motif have all been observed previously in XR variants from other species, where the cofactor specificity has been altered in favour of NADH (Fig. 5a, [58–60]). Improving the NADH/NADPH ratio can be achieved either by increasing the affinity for NADH or by decreasing the affinity for NADPH. Here, enzyme variants with improved affinity for NADH are more useful than those where the affinity for NAPDH is lost, as poor co-factor affinity negatively affects the enzyme’s xylose conversion-activity. CiXR2 belongs to the former category, as although it lacks specificity for NADPH, the xylose conversion-activity (V_max_) is maintained at levels comparable to other XRs [58–60]. This holds true also for the *S. passalidarum* XR2 (SpXR2), which just like CiXR2 contains an aspartate in the position equivalent to CtXR Asn276 and displays a NADH/NADPH ratio of 1.21 (Fig. 5a). When expressed in *S. cerevisiae*, the SpXR2 enzyme improve ethanol yields and productivity from xylose under low oxygen conditions compared to cells expressing SsXR [60]. Moreover, XR1 from *M. guilliermondii* also contains Gly275 and the Asp276, and likely displays dual co-factor specificity with high affinity for NADH. Thus, both CiXR2 and MgXR1 are new, interesting candidates to express in *S. cerevisiae* for elucidating the relation between the catalytic characteristics of the enzymes and the efficiency of xylose fermentation of lignocellulosic biomass.

Most yeast species capable of utilizing xylose as a carbon source present a single gene coding for XR, but some species including *S. passalidarum* and *M. guilliermondii* possess two XR genes. To the best of our knowledge, *C. intermedia* is the first yeast identified that has three genes encoding functional XRs, and their different expression patterns and co-factor specificities suggest that they have different physiological roles. With the genome sequenced, the possibility to develop a genome editing toolbox for this yeast arises, which would enable phenotypic characterization of XR deletion and/or overexpression strains as well as targeted genome-editing approaches for developing strains for industrial use. In fact, overexpression of CiXR2 has the potential to improve the xylose-fermentative capacity of *C. intermedia* by lowering the xylitol accumulation seen under oxygen-limited conditions [27].

## Conclusions

The non-conventional yeast *C. intermedia* can metabolize several important lignocellulosic sugars, including the abundant monosaccharide xylose that *S. cerevisiae* and many other industrial microorganisms cannot naturally ferment. The aim of the present work was to understand the genetics underlying *C. intermedia’s* capacity to ferment lignocellulosic-derived xylose and to generate knowledge that can be used to improve lignocellulose-based fermentation processes. We describe for the first time the genome analysis of the *C. intermedia* strains CBS 141442 and PYCC 4715, which revealed two strains with high overall genomic similarity yet noticeably different due to multiple chromosomal translocations. The phylogenetic analysis firmly established the species within the CTG clade of *ascomycetes* yeasts, adding to the list of xylose-fermenting yeasts with importance in biotechnology and lignocellulosic-based production of biofuels and biochemicals. Moreover, the transcriptomic analysis of strain CBS 141442 grown in high and low concentrations of glucose and xylose provides a first insight into the xylose and glucose metabolism of *C. intermedia*, and several novel genes for xylose conversion were identified. In particular, a gene encoding an XR with equal affinity for NADH and NADPH was found, and the subsequent characterization put forward this as a valuable candidate gene to express in *S. cerevisiae* to relieve its co-factor imbalance during xylose fermentation. Collectively, the results presented expand our knowledge on the metabolism of different carbon sources in yeast and enable new comparative genomic studies.

## Materials and methods

### Yeast strains and media

The *C. intermedia* strain CBS 141442 was isolated in-house from a co-culture with *S. cerevisiae* [27]. The *C. intermedia* PYCC 4715 strain, originally isolated from sewage in Oeiras, Portugal, was obtained from the Portuguese Yeast culture collection. The *S. cerevisiae* strain BY4741 *Δgre3* was used for heterologous expression of the xylose reductases, and BY4741 (haploid), CEN.PK 122MDS (diploid) and G26 (tetraploid) were used for ploidy determination.

Cells were cultured in rich medium (YPD) (20 g L^−1^ yeast extract, 10 g L^−1^ peptone and 20 g L^−1^d-glucose), in minimal mineral medium (MM) (7.5 g L^−1^ (NH_4_)_2_SO_4_, 3.5 g L^−1^ KH_2_PO_4_, 0.75 g L^−1^ MgSO_4_·7H_2_O, 2 mL L^−1^ trace metal solution, and 1 mL L^−1^ vitamin solution) [61] or on Yeast Nitrogen Base (YNB-Ura) medium plates supplemented with glucose (20 g L^−1^), (NH_4_)_2_SO_4_ (5 g L^−1^) and Complete Supplement Mixture without uracil (CSM-Ura) (1.9 g L^−1^), and agar (20 g L^−1^).

### Species determination using PCR

PCR amplification and sequencing of the Internal Transcribed Spacer (ITS) regions and the D1-D2 region of the large-subunit RNA gene was performed using primers ITS1 (5′-TCCGTAGGTGAACCTGCGG-3′), ITS4 (5′-TCCTCCGCTTATTGATATGC-3′) and NL1 (5′-GCATATCAATAAGCGGAGGAAAAG-3′) and NL4 (5′-GGTCCGTGTTTCAAGACGG-3′), respectively [18, 62]. The PCR products were sent for sequencing, and the results were blasted against the NCBI nucleotide database.

### DNA sequencing and raw data analysis

DNA isolation, library preparation, single-molecule real-time (SMRT) sequencing (Pacific Biosciences), genome assembly and annotation were performed as previously described [27]. To assess the completeness of each genome assembly, we used the CEGMA pipeline that scans assemblies for 248 highly conserved eukaryotic genes [63].

### Bioreactor cultivation and RNA sampling

For bioreactor cultivation, starter cultures were grown overnight in an orbital shaker, 200 rpm, at 30 °C. Cells were harvested and washed with sterile distilled water and the pellets were weighed. Sterile water was added to the pellets to aid resuspension in a 1:1 ratio. The inoculum volume was calculated to achieve 0.25 g L^−1^ dry weight, considering a dilution factor of 2, a cell density of ≈ 1 g mL^−1^ and a dry weight corresponding to 25% of the wet weight.

The cells were then inoculated in 400 mL of MM containing glucose (20 or 200 g L^−1^) or xylose (20 or 200 g L^−1^) in controlled stirred 1-L bioreactor vessels (DASGIP fermenter system with 8 parallel SR0700ODLS vessels, Eppendorf, Hamburg, Germany) at 30 °C. The medium pH was maintained at 5.5 with 2 M potassium hydroxide. All reactors were equipped with pH and dissolved oxygen probes (Mettler Toledo probes, Mettler Toledo, Greifensee, Switzerland) and off-gas analysers. The CO_2_ (v/v, %) and O_2_ (v/v, %) in the off-gas were monitored continuously using BlueSens gas analysers (BlueSens Gas Technology GmbH, Herten, Germany) to calculate the volumetric off-gas rates, namely the CO_2_ production rate (mmol L^−1^ h^−1^) and the O_2_ consumption rate (mmol L^−1^ h^−1^). The aeration rate was fixed at 1 Vessel Volume per Minute, with stirring at 300 rpm.

Growth was followed by measurement of OD at 600 nm in a Spectrophotometer (Genesys 20, ThermoFisher—Waltham, MA, USA). Cell samples (10 mL) were collected when the culture dissolved oxygen was 35–40% (v/v). The cells were diluted in 35 mL of very cold distilled water in a falcon tube and place it in an ice bath. Cells were then harvested by centrifugation at 5000 rpm for 5 min, at 2 °C. The pellets were resuspended in ice-cold distilled water and centrifuged again at 10,000 rpm for 5 min, at 0 °C. The pellets were frozen in liquid nitrogen and then stored at − 80 °C until extraction.

### RNA sequencing and raw data analysis

The frozen pellets were thawed in 500 μL of TRIzol (Ambion—Foster City, CA, USA) and thoroughly resuspended, and then transferred to 2-mL tubes with Lysing Matrix C (MP Biomedical—Santa Ana, CA, USA). The tubes were incubated in a FastPrep FP120 (Savant—Carlsbad, CA, USA) for five cycles, at intensity 5.5 for 30 s. Tubes were cooled on ice for a minute between cycles. Another 500 μL of TRIzol were added to each tube and vortexed thoroughly. The tubes were incubated at room temperature for 5 min and then centrifuged 10 min at 12,000 rpm, 4 °C. The supernatants were collected, chloroform was added (200 μL of chloroform per mL of supernatant) and vortexed vigorously for 30 s. The samples were incubated at room temperature for 5 min, and then centrifuged 15 min at 12,000 rpm, 4 °C. The top clear aqueous phase was collected and transferred to a new RNase-free tube. An equal amount of absolute ethanol was slowly added, mixing it as needed. Each sample was loaded into a RNeasy column (RNeasy Mini Kit, Qiagen—Hilden, Germany), further steps followed the protocol of the manufacturer. The RNA was treated with DNase in-column twice at room temperature for 30 min. The RNA was eluted with RNase-free water and the samples were stored at − 80 °C until use.

RNA samples were analysed in a TapeStation (Agilent—Santa Clara, CA, USA), and only samples with RNA integrity number above 8 were used for library preparation. Sequencing libraries were prepared from 1 μg total RNA using the TruSeq stranded mRNA library preparation kit (Cat# RS-122-2101/2102, Illumina Inc.—San Diego, CA, USA) including polyA selection. The library preparation was performed according to the manufacturers’ protocol (#15031047). The libraries were then sequenced using the HiSeq 2500 system (Illumina Inc.—San Diego, CA, USA), with paired-end 125 bp read length, and v4 sequencing chemistry. A sequencing library for the phage PhiX was included as 1% spike-in in the sequencing run. The sequencing generated 180 M of read-pairs, yielding a coverage of 5–20 M reads per condition.

The obtained read data were quality controlled using the software FastQC version 0.11.5 [64]. Following this, reads were mapped to the reference genome with the software Star version 2.5.2b [65] after using the annotation information to generate the genome index. Read counts were extracted from the BAM files by counting all reads mapping to exonic regions and merging exon counts to gene level using featurecounts [66]. Gene counts were imported into R and all subsequent analysis were done using R [67]. First the counts were normalized with weighted trimmed mean of M-values using the calcNormFactor function from the package edgeR [68]. Second, the Limma package [69] were used to transform and make data suitable for linear modelling. Finally, significant effects of growth conditions were identified using a multifactor linear model. The estimated p-values were corrected for multiple testing with the Benjamini–Hochberg procedure, and genes were considered significant if the adjusted p-values were lower than 0.05.

### Genome synteny and reciprocal read mapping

For genome synteny analysis, the two genomes where aligned using nucmer from the MUMmer package version 3.2 [70], applying the CBS 141442 assembly as reference and PYCC 4715 as query. Each aligned segment was drawn as a link from the reference to the query and each chromosome was plotted relative to its size.

For reciprocal read mapping, the CBS 141442 genome reads were mapped using BLASR v.1.3.1.127046 to the CBS 141442 assembly, with the option-*bestn 1* to only report 1 alignment [71]. The reads that mapped with a mapping quality above 1 were marked with the chromosome identity, and these marked reads were then mapped with BLASR to the PYCC 4715 assembly. The procedure was repeated by mapping the marked reads from PYCC 4715 to the CBS 141442 assembly. Here, the raw read counts were used and thus the number of reads is not always correlated with chromosome size (as for the mitochondria where many reads mapped).

### Sequence alignments, phylogenetic analysis and structural figures

BLAST search hits of the NCBI’s nucleotide and protein databases were used to supplement the sequence data generated in this study. For species phylogenetic analysis, nucleotide sequences for 10 protein coding genes (*ACT1*, *FBP1*, *PGK1*, *GPD1*, *HXK2*, *SNF1*, *HIS3*, *LEU2*, *PMA1* and *TAL1*) from the two *C. intermedia* strains and 15 other yeast species with *S. pombe* as outgroup were aligned, trimmed and concatenated. From this dataset, containing 10,652 characters, a maximum likelihood (ML) phylogenetic tree with bootstrap value 1000 was constructed using MEGA X [72, 73].

The multiple sequence alignment of the XR motifs was completed with Clustal Omega [74, 75] and was visualized with ESPRIPT [76]. The structural figures were made in Pymol (The PyMOL Molecular Graphics System, Version 2.0, Schrödinger, LLC) using the coordinates for the *C. tenuis* XR in complex with NADP^+^ (b, PDB ID: 1k8c) and NAD^+^ (c, PDB ID: 1mi3). The *C. intermedia* XR2 homology model was created with PHYRE2 [77] using the XR from *C. tenuis* as a template (PDB ID: 1k8c).

### Heatmap

The raw counts were filtered for lowly expressed genes. Genes with CPM > 3.84 in at least 12% (5/43) of samples were retained. Raw counts were converted to variance-stabilized-counts (VST) using function ‘varianceStabilizingTransformation()’ from R package ‘DESeq2’ [78]. Sample-to-sample euclidean distances were computed on the VST counts. Multi-dimensional scaling (MDS) was run on the distance matrix using R function ‘cmdscale()’. Scatterplots from MDS axes were created in R. VST counts were scaled genewise to z-scores. Z-scores were hierarchically clustered on genes and samples to generate the heatmap. The heatmap was created using R package ‘pheatmap’.

### Flow cytometry

The ploidy of *C. intermedia* CBS 141442 was investigated by flow cytometry of cells stained with Sytox green (Thermo Fischer Scientific). Exponentially growing cells were harvested and fixated in cold 70% ethanol. The cells were washed in 10 mM EDTA, pH 8.0, followed by treatment with RNase A (Thermo Fischer Scientific), 0.1 mg mL^−1^ at 37 °C for 2 h. Sytox green was added to a final concentration of 1 µM and the cells were analysed with a Guava easyCyte 8HT flow cytometry System (Merck Millipore, Darmstadt, Germany). *S. cerevisiae* strains of different ploidy were used as references.

### HPLC analysis of sugar consumption and metabolite formation

The glucose and xylose consumption and production of metabolites (glycerol, xylitol and ethanol) were analysed using HPLC (LC-4000, Jasco Inc—Easton, MD, USA) with a Rezex ROA H+ (8%) column (Phenomenex—Torrance, CA, USA). The oven temperature was set to 80 °C and the eluent was 5 mM H_2_SO_4_ at a flow rate of 0.8 mL min^−1^. A Jasco RI-4030 refractive index detector (Jasco Inc.—Easton, MD, USA) was used to quantify the sugars and the extracellular metabolites.

### Cloning of xylose reductases

The *C. intermedia* XR genes were codon optimized, with special attention given to the CTG codons, flanked with sequences for the restriction sites for BamHI and XhoI and synthesized at Eurofins Genomics (Ebersberg, Germany). The codon-optimized *S. stipitis* XR gene *XYL1_K270R* gene was obtained by PCR amplification from a plasmid developed previously in the group [79], where the forward primer included the restriction for ClaI and the reverse primer included the restriction site for the XhoI enzyme. The *C. intermedia* and *S. stipitis* genes were digested by BamHI*/*XhoI and ClaI*/*XhoI, respectively, and cloned into the plasmid p426GPD linearized with the corresponding enzymes. All constructs were sequenced and then transformed into the *S. cerevisiae* strain BY4741 *Δgre3*, using the modified lithium acetate/PEG/ssDNA methodology [80]. Positive transformants were selected for on YNB-Ura plates and confirmed with colony PCR.

### Enzymatic activity assay

Transformants were grown in shake flasks under aerobic conditions in CSM-Ura at 30 °C, 200 rpm. The cells were collected in the mid-exponential growth phase by centrifugation (5 min at 5100 rpm) and washed twice with cold water. The cell pellets were resuspended in 1 mL breaking buffer (0.1 M triethanolamine buffer (pH 7.0); 1 mM phenylmethylsulfonyl fluoride (PMSF); 0.5 mM dithiothreitol; 0.5 mM EDTA) and transferred to 2-mL tubes with Lysing Matrix C (MP Biomedical—Santa Ana, CA, USA). The tubes were incubated in a FastPrep FP120 for five cycles, at a speed of 5.5 for 30 s, with ice incubations for a minute between cycles. The supernatants were collected, and the protein concentrations were determined with Bradford reagent following the supplier instructions, using BSA as standard.

Enzyme activity was determined by following the oxidation of NAD(P)H in the microplate reader (FLUOstar Omega—BMG LabTech, Ortenberg, Germany) operating at 340 nm and 30 °C. Biological duplicates were performed. The activity was measured as reported before [81], with minor changes. The reaction was started by the addition of aldose (final concentration 300 mM) and followed for 5 min. Protein extracts were diluted to a final concentration of 400-600 μg mL^−1^ of total protein. One unit was defined as the generation of 1 μmol NAD(P) per minute. The specific enzyme activities were given in units (U) per mg protein [58]. Determination of K_m_ for NADH and NADPH was performed measuring the activity in a range of different co-factor concentrations (5–400 μM) while keeping the substrate concentration constant (300 mM).

## Supplementary information

**Additional file 1.** List of the locations and substrates where different *C. intermedia* strains have been isolated.

**Additional file 2.** GO enrichment analysis by high-throughput functional annotation for gene product properties.

**Additional file 3.** Phylogenetic tree of putative sugar transporters from *C. intermedia* CBS 141442 and other pentose-assimilating yeasts and accession numbers for *C. intermedia* putative MFS sugar transporters and known xylose-transporters from other yeast species. References for reported xylose uptake activity are included.

**Additional file 4.***C. intermedia* CBS 141442 central carbon metabolism associated proteins and corresponding accession numbers.

## Data Availability

The genome and protein datasets generated and/or analysed during the current study are available in the in the European Nucleotide Archive (ENA) with the accession numbers LT635756 to LT635763 and LT635764 to LT635771, respectively. RNA-Seq accession number is E-MTAB-6670.

## Abbreviations

ALE: Adaptive laboratory evolution
ITS: Internal transcribed spacer
MFS: Major facilitator superfamily
NAD(P)H: Nicotinamide adenine dinucleotide (phosphate)
XDH: Xylitol dehydrogenase
XR: Xylose reductase
