# Supplementary material for: Genomic and transcriptomic analysis of Candida intermedia reveals the genetic determinants for its xylose-converting capacity
Source: Biotechnol Biofuels. 2020 Mar 12;13:48. doi: 10.1186/s13068-020-1663-9 (PMC7068945; doi:10.1186/s13068-020-1663-9)
Supplement: Supplementary file 1 — Additional file 1. List of the locations and substrates where different C. intermedia strains have been isolated. [file 13068_2020_1663_MOESM1_ESM.docx]

| **Additional file 1.** Locations and substrates where different *C. intermedia* strains were isolated. | | |
| --- | --- | --- |
| **Strain number** | **Country of origin** | **Substrate of isolation** |
| ATCC 12089 | USA | Antibiotic to Physalospora tucumanensis |
| ATCC 20178 | Japan | Production of yeast biomass. US Patent 3,909,352 |
| ATCC 20404 | Japan | Process for manufacturing alcohol by fermentation. US Patent 4,472,501 |
| ATCC 28324 | Not specified | Not specified |
| ATCC 36352 | Japan | Tabuchi T, Abe M. Method for producing citric acid. US Patent 4,389,484 |
| ATCC 60381 | Japan | Soil |
| ATCC 201070 | Brazil | Human milk |
| CBS 572 | Puerto Rico | Faeces, of American woman with tropical sprue |
| CBS 2044 | Sweden | Washed bottle in brewery |
| CBS 2047 | Sweden | Washed bottle in brewery |
| CBS 2049 | South Africa | Beer |
| CBS 2291 | Norway | Throat, of healthy person |
| CBS 2879 | South Africa | Soil |
| CBS 5159 | Germany | Skin of hand |
| CBS 5310 | Brazil | Fruit |
| CBS 5311 | Brazil | Sea water |
| CBS 5460 | unknown | Musanga smithii |
| CBS 7153 | not specified | not specified, Patent |
| CBS 8414 | Netherlands | Brine bath in cheese factory |
| CBS 141442 | Sweden | Lignocellulosic hydrolysate |
| NCYC 2504 | Slovenia | Not specified |
| NCYC 2531 | not specified | Fruit preserve used in yoghurt production |
| NRRL Y-10925 | USA | Corn insect |
| Phaff 54-16 | USA | Hawiian pineapple |
| Phaff 83-7 | not specified | Culture from general foods |
| PYCC 2499 | Portugal | Sugared condensed milk |
| PYCC 4400 | Portugal | River water |
| PYCC 4667 | Portugal | Half-cooked shrimp |
| PYCC 4715 | Portugal | Sewage |
| PYCC 5186 | UK | Concentrated orange juice |
| PYCC 5238 | Portugal | Birds excrement |
| UFMG-HB-94 | Brazil | Decaying sugarcane bagasse |
