# Supplementary material for: Genomic and transcriptomic analysis of Candida intermedia reveals the genetic determinants for its xylose-converting capacity
Source: Biotechnol Biofuels. 2020 Mar 12;13:48. doi: 10.1186/s13068-020-1663-9 (PMC7068945; doi:10.1186/s13068-020-1663-9)
Supplement: Supplementary file 2 — Additional file 2. GO enrichment analysis by high-throughput functional annotation for gene product properties. [file 13068_2020_1663_MOESM2_ESM.docx]

| **Additional file 2.** GO enrichment analysis by high-throughput functional annotation for gene product properties. The genes belonging to the different clusters in the RNA-Seq heatmap were analysed using the software Blast2GO 5.2.1 software (https://www.blast2go.com/). | | | | | | | |
| --- | --- | --- | --- | --- | --- | --- | --- |
| **Cluster** | **GO group** | **(%)** |  | **Cluster** | **GO group** | **(%)** |  |
| Cluster 1 | Transmembrane transport | 9 |  | Cluster 4 | rRNA processing | 15 |  |
|  | Lipid metabolic process | 8 |  |  | Cellular response to stimulus | 12 |  |
|  | Phosphate-containing compound metabolic process | 7 |  |  | Regulation of transcription, DNA templated | 11 |  |
|  | Translation | 7 |  |  | Translation | 8 |  |
|  | Protein transport | 7 |  |  | Oxidation-reduction process | 7 |  |
|  | RNA processing | 7 |  |  | Organelle organization | 7 |  |
|  | Ribonucleoprotein complex biogenesis | 7 |  |  | Regulation of gene expression | 6 |  |
|  | Cellular protein modification process | 6 |  |  | Organic substance transport | 4 |  |
|  | Oxidation-reduction process | 5 |  |  | Cellular response to stress | 4 |  |
|  | Intracellular transport | 5 |  |  | Protein localization | 4 |  |
|  | Cellular response to stimulus | 4 |  |  | Macromolecule modification | 4 |  |
|  | Organelle organization | 4 |  |  | Regulation of cellular metabolic process | 4 |  |
|  | Regulation of gene expression | 4 |  |  | Regulation of nitrogen compound metabolic process | 3 |  |
|  | Regulation of cellular macromolecule biosynthetic process | 4 |  |  | Nitrogen component transport | 3 |  |
|  | Cellular protein localization | 4 |  |  | Cellular protein-containing complex assembly | 3 |  |
|  | RNA biosynthetic process | 4 |  |  | Small molecule metabolic process | 3 |  |
|  | ncRNA metabolic process | 3 |  |  | Phosphate-containing compound metabolic process | 1 |  |
|  | Small molecular metabolic process | 2 |  |  | Regulation of primary metabolic process | 1 |  |
|  | Regulation of nitrogen compound metabolic process | 1 |  | Cluster 5 | Transmembrane transport | 27 |  |
|  | Regulation of primary metabolic process | 1 |  |  | Regulation of transcription, DNA templated | 12 |  |
| Cluster 2 | Protein phosphorylation | 12 |  |  | Oxidation-reduction process | 11 |  |
|  | Cellular response to DNA Damage stimulus | 11 |  |  | Phosphate-containing compound metabolic process | 10 |  |
|  | Regulation of transcription by RNA pol II | 10 |  |  | Ion transport | 7 |  |
|  | Transmembrane transport | 9 |  |  | Cellular protein modification process | 7 |  |
|  | Protein transport | 9 |  |  | Organic substance transport | 6 |  |
|  | Regulation of catalytic activity | 8 |  |  | Cellular response to stress | 6 |  |
|  | Vesicle mediated transport | 8 |  |  | Nitrogen compound process | 5 |  |
|  | DNA metabolic process | 7 |  |  | Cellular localization | 3 |  |
|  | Cell communication | 4 |  |  | Cellular catabolic process | 2 |  |
|  | Organelle organization | 3 |  |  | Organonitrogen compound biosynthetic process | 2 |  |
|  | Cellular catabolic process | 3 |  |  | Small molecular metabolic process | 1 |  |
|  | Ion transport | 2 |  | Cluster 6 | Transmembrane transport | 12 |  |
|  | Response to chemical | 2 |  |  | Cellular response to stress | 12 |  |
|  | Organic substance catabolic process | 2 |  |  | Regulation of transcription, DNA templated | 11 |  |
|  | Organonitrogen compound catabolic process | 2 |  |  | Cellular amino acid metabolic process | 11 |  |
|  | Establishment of localization in cell | 2 |  |  | Oxidation-reduction process | 9 |  |
|  | Positive regulation of cellular process | 2 |  |  | Phosphate-containing compound metabolic process | 7 |  |
|  | Small molecular metabolic process | 1 |  |  | Organonitrogen compound biosynthetic process | 7 |  |
|  | Negative regulation of macromolecule metabolic process | 1 |  |  | Cellular protein modification process | 4 |  |
|  | Negative regulation of cellular metabolic process | 1 |  |  | Cellular catabolic process | 4 |  |
|  | Regulation of cellular component organization | 1 |  |  | Organelle organization | 4 |  |
|  | Negative regulation of nitrogen compound metabolism | 1 |  |  | Response to chemical | 4 |  |
| Cluster 3 | Translation | 36 |  |  | Amide biosynthetic process | 4 |  |
|  | Oxidation-reduction process | 15 |  |  | Organic substance transport | 3 |  |
|  | Transmembrane transport | 11 |  |  | Nitrogen component transport | 3 |  |
|  | Intracellular transport | 7 |  |  | Organonitrogen compound catabolic process | 3 |  |
|  | Cellular component biogenesis | 6 |  |  | Cellular component biogenesis | 2 |  |
|  | RNA metabolic process | 6 |  |  | Small molecule biosynthetic process | 1 |  |
|  | Organic substance transport | 5 |  | Cluster 7 | Protein localization | 13 |  |
|  | Macromolecule localization | 4 |  |  | Oxidation-reduction process | 11 |  |
|  | Mitochondrion organization | 4 |  |  | Transmembrane transport | 9 |  |
|  | Nitrogen compound process | 3 |  |  | Carbohydrate metabolic process | 9 |  |
|  | Small molecular metabolic process | 2 |  |  | Phosphorylation | 8 |  |
|  | Biological regulation | 2 |  |  | Cellular catabolic process | 7 |  |
|  |  |  |  |  | Regulation of transcription by RNA pol II | 7 |  |
|  |  |  |  |  | Cellular response to stress | 6 |  |
|  |  |  |  |  | Cellular protein modification process | 5 |  |
|  |  |  |  |  | Response to chemical | 5 |  |
|  |  |  |  |  | Organelle organization | 4 |  |
|  |  |  |  |  | Organic substance transport | 4 |  |
|  |  |  |  |  | Cellular localization | 4 |  |
|  |  |  |  |  | Organic substance catabolic process | 4 |  |
|  |  |  |  |  | Nitrogen component transport | 2 |  |
|  |  |  |  |  | Cellular component biogenesis | 2 |  |
|  |  |  |  |  | Small molecular metabolic process | 2 |  |
