# Supplementary material for: Genomic and transcriptomic analysis of Candida intermedia reveals the genetic determinants for its xylose-converting capacity
Source: Biotechnol Biofuels. 2020 Mar 12;13:48. doi: 10.1186/s13068-020-1663-9 (PMC7068945; doi:10.1186/s13068-020-1663-9)
Supplement: Supplementary file 3 — Additional file 3. Phylogenetic tree of putative sugar transporters from C. intermedia CBS 141442 and other pentose-assimilating yeasts and accession numbers for C. intermedia putative MFS sugar transporters and known xylose-transporters from other yeast species. References for reported xylose uptake activity are included. [file 13068_2020_1663_MOESM3_ESM.docx]

**Additional file 3.**


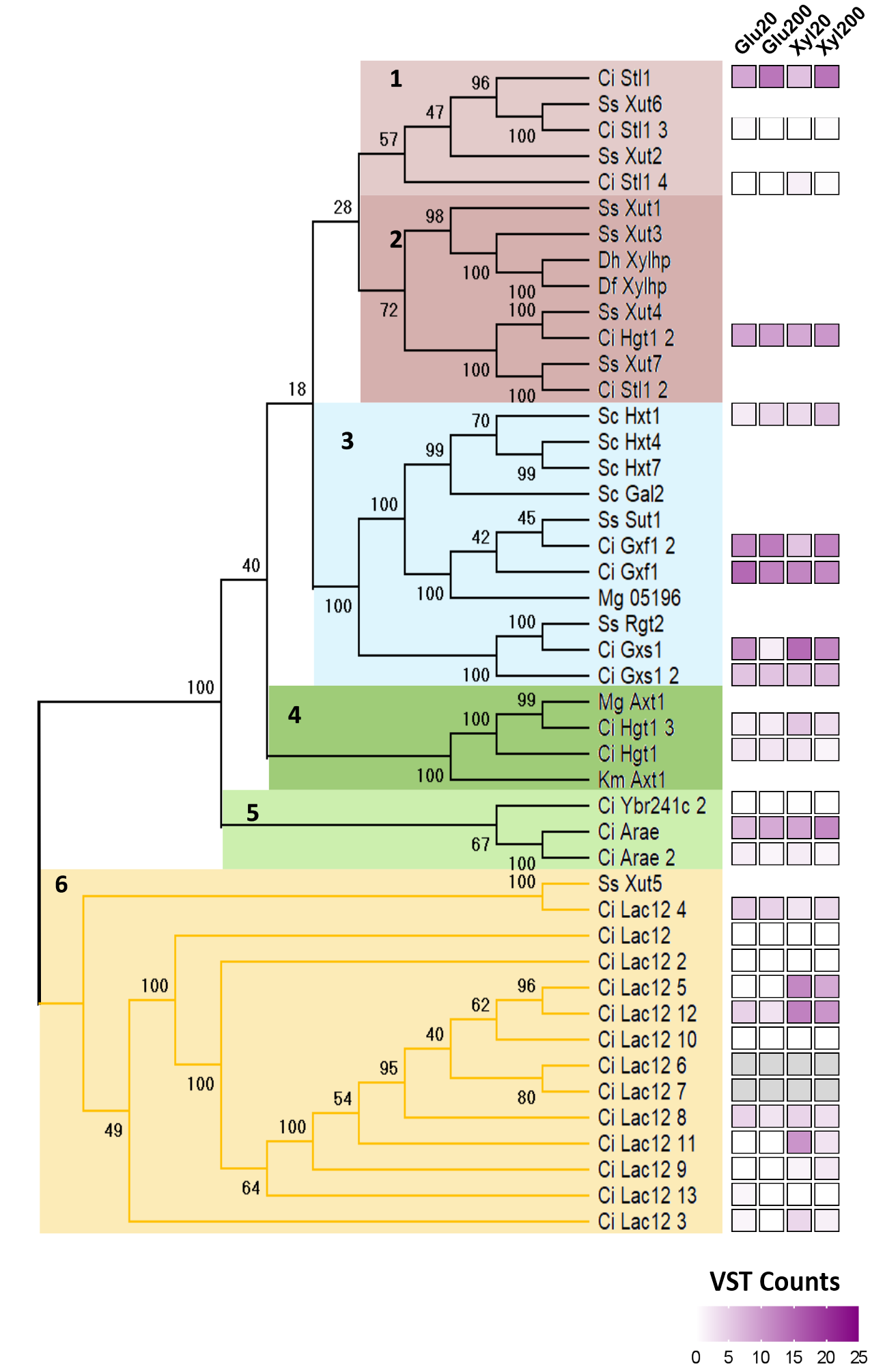


**Additional file 3.** **Phylogenetic tree of putative sugar transporters from *C. intermedia* and other pentose-assimilating yeasts**. Putative sugar transporters in the *C. intermedia* CBS 141442 genome were tracked using protein sequences from characterized yeast transporters (*S. cerevisiae* Hxt1 and Stl1; *S. stipitis* Sut1 and Xut1; *C. intermedia* Gxs1 and Gxf1) as probes. Additionally, transporters reported as capable of xylose uptake from other yeast species were added to the dataset. A total of 45 sequences were aligned, and then used to construct a ML phylogenetic tree using the maximum-likelihood method with bootstrap numbers indicated at each branching point. The accession numbers are listed in the table below. Our analysis revealed a tree with 6 distinct branches of transporters. Branches 1 and 2 contain five transporters from *C. intermedia*, some of them closely related to several of the Xut transporters from *S. stipitis* that are known to transport xylose [1-4]. The previously identified Gxf1 and Gxs1 and novel homologs Gxf1_2 and Gxs1_2 from *C. intermedia* fall into branch 3, together with Hxt1, 4 and 7 and Gal2 from *S. cerevisiae* and Sut1 from *S. stipitis*, all of which are previously shown to transport xylose but having a much higher affinity for glucose over xylose [3, 5]. Branch 5 contains five *C. intermedia* genes for transporters that show similarities to either the arabinose/xylose transporters in *M. guilliermondii* and *K. marxianus* [6] or the putative arabinose-proton symporter in *S.* *stipitis* [2], and branch 6 contains 13 *C. intermedia* genes where the corresponding proteins show homology to permeases selective for disaccharides such as lactose and cellobiose as well as the monosaccharide galactose [7]. Normalized expression of *C. intermedia* protein-encoding genes is represented in all growth conditions using variance-stabilized counts (VST). Proteins are preceded by a prefix identifying the yeast species. Sc – *Saccharomyces cerevisiae*; Ss - *Scheffersomyces stipitis*; Sp - *Spathaspora passalidarum*; Ci – *Candida intermedia*; Km – *Kluyveromyces marxianus;* Mg - *Meyerozyma guilliermondii*; Dh – *Debaryomyces hansenii*; Df - *Debaryomyces fabryi.*

| Accession numbers for *C. intermedia* CBS 141442 putative MFS sugar transporters and known xylose-transporters from other yeast species. References for reported xylose uptake activity are included. | | |
| --- | --- | --- |
| Gene | Accession Number | Reference |
| Sc Stl1 | NP_010825.3 |  |
| Sc Hxt1 | NP_011962.1 | Hamacher et al. 2002 |
| Sc Hxt4 | NP_011960.2 | Hamacher et al. 2002 |
| Sc Hxt7 | NP_010629.3 | Hamacher et al. 2002;  Young et al. 2011 |
| Sc Gal2 | NP_013182.1 | Hamacher et al. 2002;  Young et al. 2011 |
| Ss Xut1 | XP_001385583.1 | Young et al. 2011 |
| Ss Xut2 | XP_001387242.2 | Du et al. 2010 |
| Ss Xut3 | XP_001387138.1 | Young et al. 2011 |
| Ss Xut4 (Hgt3) | XP_001386715.1 | Ma et al. 2012;  Moon et al. 2013 |
| Ss Xut5 | XP_001385962.2 | Moon at al. 2013 |
| Ss Xut6 (Stl12) | XP_001386589.1 | Ma et al. 2012;  Moon et al. 2013 |
| Ss Xut7 (Stl13) | XP_001387067.1 | Moon et al. 2013 |
| Ss Sut1 | XP_001387898.1 | Katahira et al. 2008 |
| Ss Rgt2 | XP_001386588.1 | Young et al. 2014 |
| Dh Xylhp | XP_458169.1 | Young et al. 2011;  Ferreira et al. 2013 |
| Df Xylhp | AAR06925.2 | Ferreira et al. 2013 |
| Mg 05196 | XP_001482176.1 | Wang et al. 2015 |
| Mg Axt1 | XP_001482096.1 | Knoshaug et al. 2015 |
| Km Axt1 | XP_022674058.1 | Knoshaug et al. 2015 |
| Ci Hgt1 | SGZ50992.1 |  |
| Ci Hgt1_2 | SGZ51695.1 |  |
| Ci Hgt1_3 | SGZ49593.1 |  |
| Ci GXF1 | SGZ57542.1 | Leandro et a. 2006;  Young et al. 2011 |
| Ci GXF1_2 | SGZ47691.1 |  |
| Ci GXS1 | SGZ53008.1 | Leandro et a. 2006;  Young et al. 2011 |
| Ci GXS1_2 | SGZ50173.1 |  |
| Ci Stl1 | SGZ58446.1 |  |
| Ci Stl1_2 | SGZ47012.1 |  |
| Ci Stl1_3 | SGZ46314.1 |  |
| Ci Stl1_4 | SGZ57759.1 |  |
| Ci Lac12 | SGZ47224.1 |  |
| Ci Lac12_2 | SGZ49661.1 |  |
| Ci Lac12_3 | SGZ48118.1 |  |
| Ci Lac12_4 | SGZ57765.1 |  |
| Ci Lac12_5 | SGZ55027.1 |  |
| Ci Lac12_6 | SGZ54820.1 |  |
| Ci Lac12_7 | SGZ51011.1 |  |
| Ci Lac12_8 | SGZ47318.1 |  |
| Ci Lac12_9 | SGZ57769.1 |  |
| Ci Lac12_10 | SGZ54727.1 |  |
| Ci Lac12_11 | SGZ55091.1 |  |
| Ci Lac12_12 | SGZ51023.1 |  |
| Ci Lac12_13 | SGZ47317.1 |  |
| Ci Ybr241c_2 | SGZ47420.1 |  |
| Ci Arae | SGZ57929.1 |  |
| Ci Arae_2 | SGZ47799.1 |  |

**References**

1. Moon J, Lewis Liu Z, Ma M, Slininger PJ: **New genotypes of industrial yeast *Saccharomyces cerevisiae* engineered with YXI and heterologous xylose transporters improve xylose utilization and ethanol production**. *Biocatal Agric Biotechnol* 2013, **2**(3):247-254.

2. Ma M, Liu ZL, Moon J: **Genetic Engineering of Inhibitor-Tolerant *Saccharomyces cerevisiae* for Improved Xylose Utilization in Ethanol Production**. *BioEnergy Res* 2012, **5**(2):459-469.

3. Young E, Poucher A, Comer A, Bailey A, Alper H: **Functional survey for heterologous sugar transport proteins, using Saccharomyces cerevisiae as a host**. *Appl Environ Microbiol* 2011, **77**(10):3311-3319.

4. Du J, Li S, Zhao H: **Discovery and characterization of novel d-xylose-specific transporters from Neurospora crassa and Pichia stipitis**. *Mol Biosyst* 2010, **6**(11):2150-2156.

5. Hamacher T, Becker J, Gardonyi M, Hahn-Hagerdal B, Boles E: **Characterization of the xylose-transporting properties of yeast hexose transporters and their influence on xylose utilization**. *Microbiology* 2002, **148**(Pt 9):2783-2788.

6. Knoshaug EP, Vidgren V, Magalhães F, Jarvis EE, Franden MA, Zhang M, Singh A: **Novel transporters from *Kluyveromyces marxianus* and *Pichia guilliermondii* expressed in *Saccharomyces cerevisiae* enable growth on L-arabinose and D-xylose**. *Yeast* 2015, **32**(10):615-628.

7. Rigamonte TA, Silveira WB, Fietto LG, Castro IM, Breunig KD, Passos FML: **Restricted sugar uptake by sugar-induced internalization of the yeast lactose/galactose permease Lac12**. *FEMS yeast research* 2011, **11**(3):243-251.
