# Supplementary material for: Genomic and transcriptomic analysis of Candida intermedia reveals the genetic determinants for its xylose-converting capacity
Source: Biotechnol Biofuels. 2020 Mar 12;13:48. doi: 10.1186/s13068-020-1663-9 (PMC7068945; doi:10.1186/s13068-020-1663-9)
Supplement: Supplementary file 4 — Additional file 4.C. intermedia CBS 141442 central carbon metabolism associated proteins and corresponding accession numbers. [file 13068_2020_1663_MOESM4_ESM.docx]

| **Additional file 4.** *C. intermedia* CBS141442 central carbon metabolism associated proteins and corresponding accession numbers. | |
| --- | --- |
| **Protein** | **Accession Number** |
| Xyl1 | SGZ54790.1 |
| Xyl1_2 | SGZ56686.1 |
| Xyl1_3 | SGZ50191.1 |
| Xyl2 | SGZ50894.1 |
| Xks1 | SGZ54087.1 |
| Rpe1 | SGZ53240.1 |
| Dor14 | SGZ57171.1 |
| Sol1 | SGZ54257.1 |
| G6pd | SGZ52322.1 |
| Glk1 | SGZ46694.1 |
| Hxk1 | SGZ54199.1 |
| Hxk2 | SGZ54879.1 |
| Hxk2_2 | SGZ54881.1 |
| Rki1 | SGZ54330.1 |
| Pgi1 | SGZ54794.1 |
| Tkt1 | SGZ49130.1 |
| Tal1 | SGZ46696.1 |
| Fbp1 | SGZ55829.1 |
| Fba1 | SGZ54139.1 |
| Tpi1 | SGZ54439.1 |
| Tdh1 | SGZ53670.1 |
| Gpd3 | SGZ56908.1 |
| Pgk1 | SGZ56351.1 |
| Gpm1 | SGZ55658.1 |
| Gpma | SGZ50474.1 |
| Eno1 | SGZ51012.1 |
| Eno2 | SGZ47884.1 |
| Pyk1 | SGZ49091.1 |
